# Supplementary material for: FastqPuri: high-performance preprocessing of RNA-seq data
Source: BMC Bioinformatics. 2019 May 3;20:226. doi: 10.1186/s12859-019-2799-0 (PMC6500068; doi:10.1186/s12859-019-2799-0)
Supplement: Supplementary file 2 — Archive of FastqPuri. Archive containing all files needed to install and run FastqPuri v1.0.6. Date stamp March 22, 2019. (GZ 47,819 kb) [file 12859_2019_2799_MOESM2_ESM.gz › FastqPuri-1.0.6/html/struct__ad__seq.html]

FastqPuri: \_ad\_seq Struct Reference


FastqPuri

Public Attributes |
List of all members

\_ad\_seq Struct Reference

stores an adapter entry
More...

`#include <adapters.h>`

|  |  |
| --- | --- |
| Public Attributes | |
| int | L |
|  | |
| char | seq [READ\_MAXLEN] |
|  | |
| int | Lpack |
|  | |
| int | Lpack\_sh |
|  | |
| unsigned char | pack [(READ\_MAXLEN+1)/2] |
|  | |
| unsigned char | pack\_sh [(READ\_MAXLEN+1)/2] |
|  | |

## Detailed Description

stores an adapter entry

## Member Data Documentation

## ◆ L

int \_ad\_seq::L

length of the adapter

## ◆ Lpack

int \_ad\_seq::Lpack

length of the packed sequence as is

## ◆ Lpack\_sh

int \_ad\_seq::Lpack\_sh

length of the shifted packed sequence

## ◆ pack

unsigned char \_ad\_seq::pack[(READ\_MAXLEN+1)/2]

packed sequence

## ◆ pack\_sh

unsigned char \_ad\_seq::pack\_sh[(READ\_MAXLEN+1)/2]

packed shifted sequence

## ◆ seq

char \_ad\_seq::seq[READ\_MAXLEN]

adapter sequence

---

The documentation for this struct was generated from the following file:

- include/adapters.h


---

Generated on Mon Mar 19 2018 23:42:01 for FastqPuri by  

 1.8.14
